# COVID-19 spike polypeptide vaccine reduces the pathogenesis and viral infection in a mouse model of SARS-CoV-2

**DOI:** 10.3389/fimmu.2023.1098461

**Published:** 2023-03-03

**Authors:** Yasmin Hisham, Sun-Min Seo, Sinae Kim, Saerok Shim, Jihyeong Hwang, Eun-Seon Yoo, Na-Won Kim, Chang-Seon Song, Hyunjhung Jhun, Ho-Young Park, Youngmin Lee, Kyeong-Cheol Shin, Sun-Young Han, Je Kyung Seong, Yang-Kyu Choi, Soohyun Kim

**Affiliations:** ^1^Laboratory of Cytokine Immunology, Department of Biomedical Science and Technology, Konkuk University, Seoul, Republic of Korea; ^2^Department of Laboratory Animal Medicine, College of Veterinary Medicine, Konkuk University, Seoul, Republic of Korea; ^3^College of Veterinary Medicine, Konkuk University, Seoul, Republic of Korea; ^4^Food Industry Infrastructure Team, Korea Food Research Institute, Wanju, Republic of Korea; ^5^Research Group of Functional Food Materials, Korea Food Research Institute, Wanju, Republic of Korea; ^6^Department of Medicine, Pusan Paik Hospital, Inje University College of Medicine, Busan, Republic of Korea; ^7^Center for Respiratory Disease, College of Medicine, Yeungnam University, Daegu, Republic of Korea; ^8^College of Pharmacy and Research Institute of Pharmaceutical Sciences, Gyeongsang National University, Jinju, Gyeongsangnam, Republic of Korea; ^9^Laboratory of Developmental Biology and Genomics, Research Institute for Veterinary Science, and BK21 PLUS Program for Creative Veterinary Science Research, College of Veterinary Medicine, Seoul National University, Seoul, Republic of Korea; ^10^Korea Mouse Phenotyping Center, Interdisciplinary Program for Bioinformatics, and BioMAX Institute, Seoul National University, Seoul, Republic of Korea

**Keywords:** SARS-CoV-2, pathogenesis, COVID-19 vaccine, spike polypeptide, *in vivo* mouse model

## Abstract

The SARS-CoV-2 coronavirus, which causes a respiratory disease called COVID-19, has been declared a pandemic by the World Health Organization (WHO) and is still ongoing. Vaccination is the most important strategy to end the pandemic. Several vaccines have been approved, as evidenced by the ongoing global pandemic, but the pandemic is far from over and no fully effective vaccine is yet available. One of the most critical steps in vaccine development is the selection of appropriate antigens and their proper introduction into the immune system. Therefore, in this study, we developed and evaluated two proposed vaccines composed of single and multiple SARS-CoV-2 polypeptides derived from the spike protein, namely, vaccine A and vaccine B, respectively. The polypeptides were validated by the sera of COVID-19-vaccinated individuals and/or naturally infected COVID-19 patients to shortlist the starting pool of antigens followed by *in vivo* vaccination to hACE2 transgenic mice. The spike multiple polypeptide vaccine (vaccine B) was more potent to reduce the pathogenesis of organs, resulting in higher protection against the SARS-CoV-2 infection.

## 1 Introduction

The recent and ongoing pandemic named COVID-19 caused by the coronavirus SARS-CoV-2 that first emerged in late 2019 continues to claim over 6 billion positive cases with higher than 6.5 million deaths (1). Symptoms are relatively similar to common cold symptoms and range from mild to severe conditions. These symptoms include coughing, shortness of breath, fatigue, and fever; the elderly especially those who have comorbidities such as hypertension, obesity, or diabetes are at higher risk for serious illness. In addition, another complication associated with SARS-CoV-2 is the development of a severe COVID-19-related “cytokine storm”, possibly due to dysregulation of the IFN-I response that causes a serious condition known as acute respiratory distress syndrome (ARDS) (2–7). To note, among all shown SARS-CoV-2 variants, the Omicron variant outbreak was the highest wave worldwide (8–10).

Combining immunization with non-pharmaceutical interventions is the greatest approach to control a pandemic. Therefore, multiple vaccines against COVID-19 have been developed at an exceptional rate, delivering billions of doses worldwide and significantly reducing the number of deaths from the COVID-19 disease (11, 12). Although several vaccines have been approved, the pandemic is not over yet, as evidenced by the ongoing global pandemic since none of the commercially available vaccines is entirely effective to prevent COVID-19. Furthermore, a series of severe cases of COVID-19 among people who had already received two doses of the Pfizer vaccine were reported in Israel in late July and early August 2021, questioning the level of effectiveness of the vaccine (13). A recent analysis shows that the COVID-19 pandemic may end in 2022, but then again COVID-19 will be two times more lethal than seasonal flu by 2023 (14). Another analysis that was performed by the British government assumed that this pandemic could be over either by 2022/2023 or by 2023/2024, or may last till 2026 (15). Both assumptions signify the need for a proper effective vaccine against SARS-CoV-2.

The major transgene or its fragments thereof that are currently primarily focused on vaccine development for COVID-19 are the spike protein of SARS-CoV-2, especially the receptor-binding domain (RBD). Therefore, consideration of the variants and the mutational events of SARS-CoV-2 should not be neglected especially for the RBD region (16–20). Moreover, the spike protein facilitates viral entry into cells as it is located on the virion surface and is believed to bind to the human angiotensin-converting enzyme 2 (hACE2) receptor, making it susceptible to humoral antibody immune responses, thus considered a promising immunogen. Furthermore, data indicate that the spike protein is the primary target of neutralizing antibodies and some of identified neutralizing antibodies were applied as therapeutic neutralizing antibodies in different clinical trial phases such as Celltrion (NCT04602000) and Regeneron (NCT04425629, NCT04426695, and NCT04452318) (21, 22).

Among the vaccine types, subunit vaccines that are composed of viral proteins or protein fragments offer stably expressed, conformationally native antigenic peptides and high-throughput, scalable solutions. The most used platforms in designing the new vaccines for SARS-CoV-2 were mRNA vaccine-based and, to a lesser extent, DNA vaccine-based platforms (23). Still, although these vaccines restrict the severe cases of COVID-19 infections and relatively reduce the spreading, drawbacks including safety and immunogenicity, long-term efficacy, and stability especially for RNA, as it is highly susceptible to degradation, are among the challenges hindering vaccine development (24). Compared to these platforms, peptide-based vaccines exhibit superior properties and guarantee cytolytic T-cell induction and memory B-cell formation (25–27). Therefore, and in order not to depend on the transcriptional and/or translation machinery (peptide production) of the body and its variation among individuals, we chose to use the peptide-based vaccine platform.

Moreover, other than DNA and mRNA vaccines, subunit (peptide) vaccines guarantee to preserve the required conformation and its final concentration (28–31). Four used antigen polypeptides were selected by the structure and immunogenicity of spike protein (16, 32–34). In addition, polypeptide vaccines are easier and cheaper to manufacture on a large scale than mRNA vaccines and do not need ultra-cold storage. This may help get more vaccines to undeveloped parts of the world like Africa where vaccination rates are very low. Nevertheless, while vaccination remains the most important strategy to end the pandemic, achieving global vaccination coverage remains a major hurdle. In this context, we examined that the selected polypeptides of the COVID-19 spike were validated by sera of vaccinated individuals and infected patients following *in vivo* vaccination using the hACE2 transgenic mouse (TG) model of COVID-19 (35). Here, we report the result of two vaccines: vaccines A and B composed of single SARS-CoV-2 and multiple SARS-CoV-2 polypeptides, respectively, derived from the SARS-CoV-2 spike protein. Vaccine B sufficiently reduced the pathogenesis of different organs, resulting in protection of hACE2 TG mice from SARS-CoV-2 infection.

## 2 Materials and methods

### 2.1 Cloning, expression, and purification of polypeptides (antigens)

Polypeptide antigens of spike protein were cloned, expressed, and purified as described earlier (17, 36). Briefly, spike cDNA corresponding to polypeptide antigens were cloned into a pET21a vector (Takara, Shiga, Japan), followed by PCR, then PCR products were ligated into an expression vector using suitable restriction enzymes (Takara, Shiga, Japan). The positive clone containing the polypeptide cDNA insert was confirmed by analysis of their respective DNA sequencing (Cosmogen, Seoul, Korea). Next, expression vectors were transformed into BL21-CodonPlus (Stratagene, San Diego, CA, USA) through a heat-shock technique. After collecting the expressed polypeptides, they were purified using their 6 × his-tag at the C-terminus by TALON^®^ Magnetic Beads (Takara) followed by HPLC purification. Their concentrations were verified *via* silver staining and Bradford assay.

### 2.2 Viral antibody testing and neutralization assay

Purified spike antigens were assessed for their neutralizing ability using serum samples from SARS-CoV-2-vaccinated and naturally infected people, which were approved by the Institutional Review Board of Yeungnam University Medical Center, Korea (approval no. 2020-07-063) (17). Homemade enzyme-linked immunosorbent assay (ELISA) was used to detect neutralizing antibodies (anti-SARS-CoV-2 antibodies) within human sera against a list of purified spike antigens, which was used to coat max-flat-bottom 96-well plates at a final concentration of 1 µg/ml and kept at 4°C 1 day before the assay. The next day (the day of the assay), the plates were washed three times with phosphate-buffered saline and 0.1% Tween (PBS-T) and blocked with 200 μl/well 2% BSA for 1 h at room temperature (RT), followed by washing with PBS-T three times, and then incubated with serially diluted serum samples for 2 h at RT. Next, the plates were washed three times with PBS-T, incubated on a rocker for 0.5 h at RT with antibody-HRP, washed three times with PBS-T, and incubated with TMP-substrate 100 μl/well for 20 min at RT followed by 100 μl/well of stop solution. The ELISA plate was read at 450 nm on a microplate reader. The same ELISA steps were used for the titration assay of mice serum.

### 2.3 Vaccine formulation, mice vaccinations, and infection

All the animal experiments were approved by the Institutional Animal Care and Use Committee (IACUC) at Konkuk University. Two vaccines, vaccine A and vaccine B, were designated among the purified spike antigens. Both were injected twice subcutaneously with 2-week intervals; the first injection (on day 21) was formulated with a complete adjuvant, and the second (on day 7) was formulated with an incomplete adjuvant. Antigens and adjuvants (Freund’s adjuvant, a known solution of antigen emulsified in mineral oil used as an immunopotentiation; both complete and incomplete adjuvants were used: the complete adjuvant is made of inactivated and dried mycobacteria, and the incomplete adjuvant lacks the mycobacterial components) were mixed in a 1:1 ratio to the mentioned final concentration. Male mice of K18-hACE2 TG at 7 weeks of age were used; each group was injected with a dose of 2 μg per mouse, and all mice were preserved with food and water and weighted and monitored daily. The first group was vaccinated with vaccine A followed by those infected/challenged with SARS-CoV-2 virus (n = 5); the second group was vaccinated with vaccine B followed by those infected/challenged with SARS-CoV-2 (n = 5); and the control group was only infected/challenged with SARS-CoV-2 (n = 5), 1 × 10^5^. The median tissue culture infectious dose (TCID_50_) of SARS-CoV-2 virus (NCCP 43326) was given intranasally at day 0. The viral infection of SARS-CoV-2 by real-time RT-PCR was performed according to the guidelines of Korea Centers for Disease Control & Prevention (KCDC&P). Next, sera were collected on day 6 or 7, and all sera were kept at 4°C until use.

### 2.4 Mouse experiment for vaccine evaluation

The following elements were checked to evaluate changes and compare the three groups of mice: mouse weight, mouse activity, and survival rate until 7 dpi. Moreover, lung, spleen, and small intestine tissue excisions from sacrificed mice on day 7 were used for histopathological score measurements. Virus titer was measured for lung tissues, and tissue weight/body weight was measured for lung tissues.

### 2.5 Histopathological analysis of lung, spleen, and small intestine

Lung, spleen, and small intestine organs from sacrificed mice were collected on day 7 after infection. The collected tissues were fixed using 4% paraformaldehyde, paraffin-embedded, cut into sections equally, and stained with hematoxylin–eosin (H&E) staining for detection of histopathological changes. Inflammation, edema, and bronchiolitis lesions were measured for lung tissues. Spleen atrophy of the white pulp was measured in the spleen. The number of goblet cells was measured in the small intestine.

### 2.6 Statistical analysis

Statistical analysis was completed using Prism 8.0 (GraphPad Software). One-way ANOVA or two-way ANOVA, followed by Tukey’s *post-hoc* correction, was used; P-value <0.05 was considered significant.

## 3 Results

### 3.1 Identifying potential protective antigens using serum samples from vaccinated and naturally infected patients

**Figure 1:**
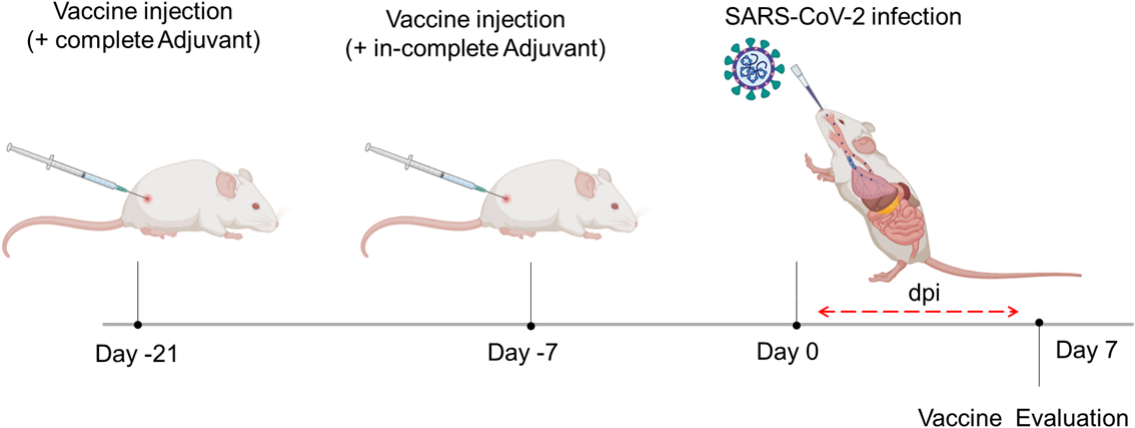
Schematic drawing showing the experimental design of the vaccination. K18-hACE2 TG mice were injected twice with 2 μg or either vaccine A or vaccine B subcutaneously at intervals of 2 weeks. After a week of a second injection, mice were infected with SARS-CoV-2 (10^5^ PFU) through the intranasal route, and 7 days postinfection changes were assessed.

Various antigens/polypeptides of the spike protein were constructed and purified to be evaluated as a vaccine candidate. These antigens were checked for their ability to bind to neutralizing antibodies within the human sera of either vaccinated people or patients naturally infected with the SARS-CoV-2 virus. Among them, four antigens summarized in **Table 1** were selected for further experiments. These antigens were used as a SARS-CoV-2 vaccine and to compare whether one antigen is sufficient to offer protection similar to multiple antigens. We used two vaccines as follows: vaccine A is composed of a single antigen-1 (Ag1), whereas vaccine B is a mixture of four antigens (Ag1, Ag2, Ag3, and Ag4). The scheme of the designed experiments is summarized in **Figure 1**. Briefly, mice were immunized with two different vaccines (two groups each with either vaccine A or vaccine B) subcutaneously in a final dose of 2 μg and scheduled within 3 weeks prior to infection as the first injection was at day 21 and the second injection was at day 7 (2-week interval), and then mice were infected with 10^5^ PFU of SARS-CoV-2 virus at day 0, sera were collected, and mice were necropsied at day 7.

**Table 1:** Amino acid sequence of four spike antigens.

Antigen ID	Antigen Sequence (a a)	Length (aa)
**Ag1 (14-134)**	**M** QCVNLTTRTQLPPANNLDSKVGGNYNYLYRLFRKSNLKPFERDISTEIYQAGSTPCNGVEGFNCYFPLQSYGFQPTNGVGYQPYRVVVLSFELLHAPATVCGPKKSTNLVKNKCVNFNFNG**LE**HHHHHH*	**123aa**
**Ag2 (358-683)**	**M**ISNCVADYSVLYNSASFSTFKCYGVSPTKLNDLCFTNVYADSFVIRGDEVRQIAPGQTGKIADYNYKLPDDFTGCVIAWNSNNLDSKVGGNYNYLYRLFRKSNLKPFERDISTEIYQAGSTPCNGVEGFNCYFPLQSYGFQPTNGVGYQPYRVVVLSFELLHAPATVCGPKKSTNLVKNKCVNFNFNGLTGTGVLTESNKKFLPFQQFGRDIADTTDAVRDPQTLEILDITPCSFGGVSVITPGTNTSNQVAVLYQDVNCTEVPVAIHADQLTPTWRVYSTGSNVFQTRAGCLIGAEHVNNSYECDIPIGAGICASYQTQTNSPRR**LE**HHHHHH*	**326aa**
**Ag3 (507-683)**	**M**PYRVVVLSFELLHAPATVCGPKKSTNLVKNKCVNFNFNGLTGTGVLTESNKKFLPFQQFGRDIADTTDAVRDPQTLEILDITPCSFGGVSVITPGTNTSNQVAVLYQDVNCTEVPVAIHADQLTPTWRVYSTGSNVFQTRAGCLIGAEHVNNSYECDIPIGAGICASYQTQTNSPRR**LE**HHHHHH*	**179aa**
**Ag4 (1040-1213)**	**M**VDFCGKGYHLMSFPQSAPHGVVFLHVTYVPAQEKNFTTAPAICHDGKAHFPREGVFVSNGTHWFVTQRNFYEPQIITTDNTFVSGNCDVVIGIVNNTVYDPLQPELDSFKEELDKYFKNHTSPDVDLGDISGINASVVNIQKEIDRLNEVAKNLNESLIDLQELGKYEQYIKWP**LE**HHHHHH*	**176aa**

The list of potential vaccine candidates of spike protein and their IDs, amino acid sequence, and length. Amino acids in black bold emphasis represent the starting residue. M, methionine. Amino acids in red are the Histidine tags at the C-terminus; red asterisk symbol (*) represents the stop codon. The underlined values showed the immunogenic part of the antigen (epitope/motif).

**Figure 2:**
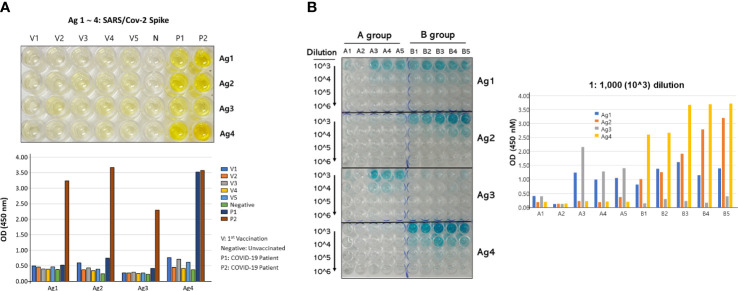
Antibody titers of serum against the four antigens. **(A)** ELISA plate showed the binding of the antigens with antibodies from sera of vaccinated (V1∼V5), negative (N), and naturally infected patients (P1/2) (upper panel), and their numerical OD_450_ values (lower panel). The patient 1 and 2 COVID-19 neutralizing indexes were 20 and 640, respectively. **(B)** ELISA plate showed the titration results of the antigens with antibodies formed in the two vaccinated mice groups (left) and their numerical OD_450_ values (right). The antibody titration was performed by serial dilution from 1:1,000 (10^3^) to 1:1,000,000 (10^6^). All vaccinated people included in this study were vaccinated by Pfizer mRNA vaccine.

Two-step purified recombinant antigens were visualized by 10% SDS-PAGE and silver staining (**Supplementary Figure 1**). These four antigens were selected to be examined as vaccine candidates because of their highest binding ability (higher OD_450_) with vaccinated or naturally infected human serum samples. COVID-19 patient 2’s resulting titer against all four antigens was high and tightly associated with the COVID-19 neutralizing index 640 (**Figure 2A**). However, the vaccinated human sera exhibited very low titers compared to naturally infected patients’ sera. In addition, the vaccinated mouse sera were examined for their titers using the four antigens (**Figure 2B**). In general, the multiple spike polypeptide (vaccine B)-immunized group exhibited higher titers compared with the single polypeptide (vaccine A)-immunized group.

### 3.2 Evaluation of survival, weight, and activity changes in mice after SARS-CoV-2 infection

**Figure 3:**
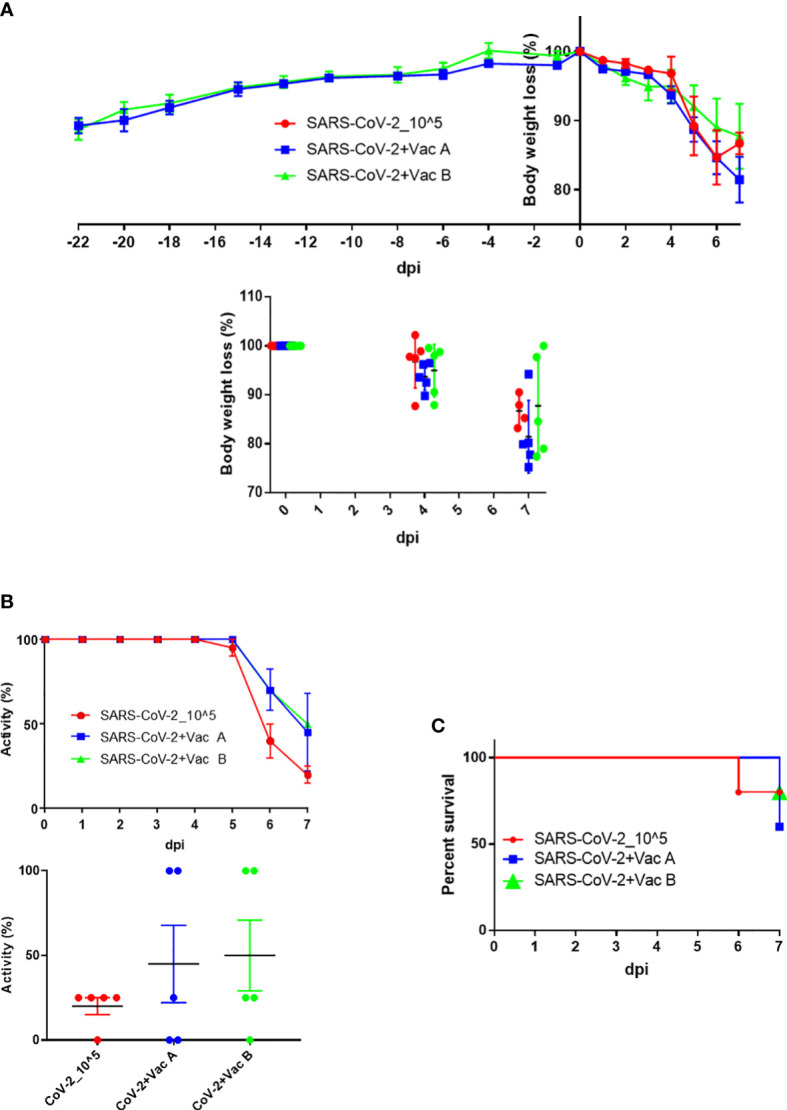
Body weight, activity, and survival changes in SARS-CoV-2-vaccinated and infected mice. **(A)** Weight changes starting from injection time (upper panel) and/or while infection (lower panel) plotted as percent change to compare the weight change of three groups (each group has n = 5); both the SARS-CoV-2-infected group (vaccine A, and vaccine B) and the vaccine-treated groups were autopsied; thus, mice’s body weights were monitored until 7 days postinfection (dpi). **(B)** Activity changes from day 0 up to day 7 postinfection (upper panel), and the overall activity changes (lower panel) plotted as percent change to compare the activity change of three groups after infection (each group has n = 5); both the SARS-CoV-2-infected group and the vaccine-treated groups (vaccine A and vaccine B). **(C)** The survival of mice was monitored every day until mice of the three groups were autopsied on day 7 dpi. Means with SD are presented, and there was no significance among groups.

After the vaccination schedule was performed as shown in **Figure 1**, the three groups of K18-hACE2 TG mice were intranasally infected and on day 7 sacrificed for collection of organ tissue and blood samples. Prior to and postinfection, the weight of mice was monitored for the two groups that have been vaccinated, that is, those vaccinated with vaccine A and those vaccinated with vaccine B, till the day of infection in which there was no difference in the body weight of these two groups. From day 0 up to day 7 postinfection, the three groups—the control group (infected only) was added—were compared for their body weight changes as percent change (**Figure 3A**, upper panel). At 6 dpi, both the SARS-CoV-2-infected group and the vaccine A-administrated group showed a body weight loss of nearly 15%, whereas the vaccine B-administrated group revealed an 11% loss of body weight. From 4 to 7 dpi, three mice of the vaccine-administered groups (one from the vaccine-A group and two from the vaccine-B group) recovered their weight after weight loss (**Figure 3A**, bottom panel). Although there was no statistical significance between the three groups in body weight changes, the vaccine B-administrated group demonstrated the lowest body weight loss and improved recovery (**Supplementary Figure 2**).

In addition, the activity of the three groups was observed and the changes were summarized as percentages (**Figure 3B**, upper panel). The activity in the SARS-CoV-2-infected group started to decrease at 5 dpi and continuously worsened leading to a moribund state at 7 dpi, whereas in the vaccine-administered group, the activity decreased from 6 dpi and then continued to decrease leading to a moribund status in some mice at 7 dpi. Moreover, the activity of two mice in the vaccine-A group as well as two mice in the vaccine-B group remained 100% up to 7 dpi, and a moribund status was observed in two mice of the vaccine-A group but one mouse of the vaccine-B group (**Figure 3B**, bottom panel). As indicated also in the survival outcomes that were monitored up to 7 dpi, all mice were sacrificed on day 7 (**Figure 3C**). There was no significant difference between the three groups in the tested measurements; body weight changes, activity, and survival; however, the mice of group vaccine-B showed the most promising results.

### 3.3 SARS-CoV-2 titer in lung and histopathological changes in infected mice with and without vaccine

**Figure 4:**
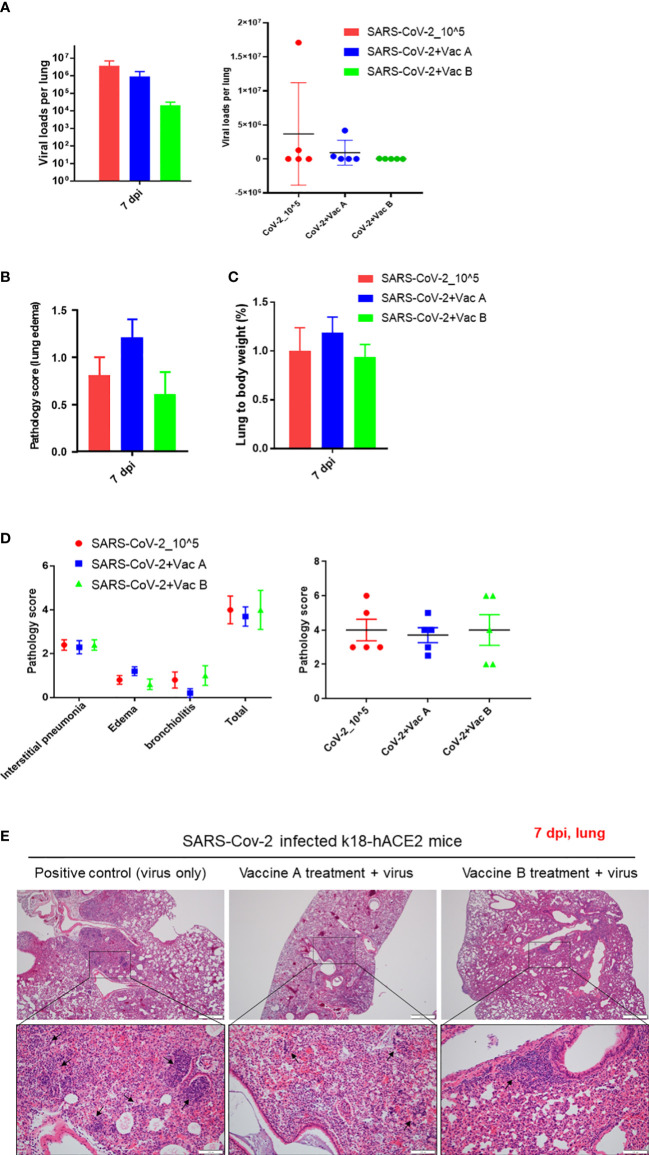
Pathological changes of K18-hACE2 TG mouse lungs after SARS-CoV-2 infection with and without vaccine. **(A)** The viral load measurements in the three groups of mice; vaccine A-administered group, vaccine B-administered group, and SARS-CoV-2-infected group (each n = 5) represented as cumulative viral load in each group in the *left* bar graph. The viral load measurements showed individual viral load in each mouse within the three groups of mice as represented in the *right* graph. Means with SD are presented, and there was no significance among groups. **(B)** Histopathological score of changes in lung edema. **(C)** Lung to body weight %. **(D)** Total pathology score. **(E)** Histopathology of mouse lungs after being paraffin-embedded, cut, and stained with hematoxylin–eosin (H&E). Lung granulomas are indicated in black arrows. Scale bars 50 µm (upper line) and100 µm (lower line).

The three groups of mice were sacrificed at 7 dpi, and the viral titers and histopathologic changes were evaluated. In the lung, both viral titers and histopathologic changes are as shown in **Figure 4**. The vaccine-B group had a markedly lower viral titer (2.1 × 10^4^) compared with those of SARS-CoV-2 infection only (3.7 × 10^7^). Also, the virus titer of the vaccine-A group (9.2 × 10^5^) showed a decrease compared with the SARS-CoV-2-infected group (**Figure 4A**), although there was no statistically significant difference. On the other hand, histopathological changes in the lungs of mice of the vaccine B-administered group were not greatly improved compared with the group of SARS-CoV-2-infected mice but was not worsened as in the vaccine A-administered group (**Figures 4B–E**). The total histopathological score showed a non-significant difference between the three groups (**Figure 4D**). However, for lung edema (**Figure 4B**), the vaccine A-administered mice showed higher edema compared with SARS-CoV-2-infected mice and vaccine B-administered mice (lowest edema score). In addition, out of five mice, two mice of the vaccine B-administered group revealed the lowest histopathological score when comparing all 15 mice of the three groups (**Figure 4D**, right panel). These quantitative histopathological scores were confirmed with the histopathological analysis of the mice’s lungs (**Figure 4E**). Lung granulomas are highly formed in the mice of SARS-CoV-2 infection, followed by the vaccine A-administered group, and the least granuloma formation was in the vaccine B-administered group. Thus, taking all together, this suggests that vaccine B confers a promising vaccine nomination as it showed lower viral titers and histopathological scores.

### 3.4 Histopathological changes in vaccinated and non-vaccinated mice after SARS-CoV-2 infection

**Figure 5:**
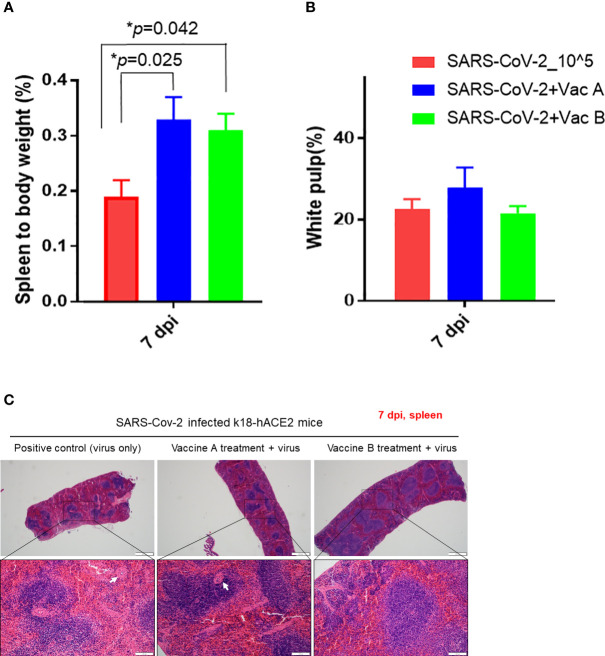
Pathological changes of K18-hACE2 TG mouse spleens after SARS-CoV-2 infection with and without vaccines. **(A)** Spleen to body weight %. **(B)** White pulp atrophy %. **(C)** Histopathology of mouse spleens after being paraffin-embedded and processed for hematoxylin–eosin (H&E) staining. Splenic white pulp atrophy is indicated in white arrows. Scale bars 50 µm (upper line) and100 µm (lower line). *p < 0.05.

**Figure 6:**
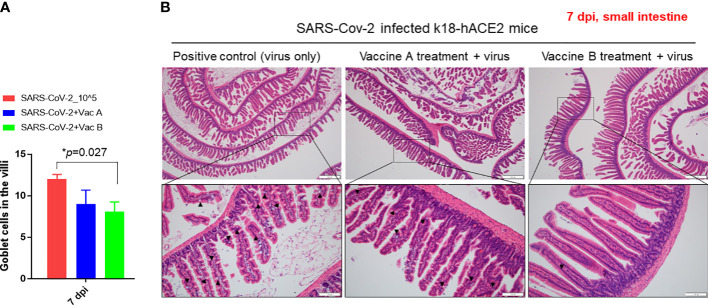
Pathological changes of K18-hACE2 TG mouse small intestines after SARS-CoV-2 infection, with and without vaccines. **(A)** Bar graph showing the number of goblet cells in villi in the three groups of mice. **(B)** Histopathology of mouse small intestine after being paraffin-embedded and stained with hematoxylin–eosin (H&E). Goblet cells are indicated in black arrows. Scale bars 50 µm (upper line) and 100 µm (lower line). *p < 0.05.

We investigated the histopathological changes in the spleen and small intestine, other than the lung, to evaluate the degree of spreading of the infection and how vaccines impact these organs (**Figures 5**, **6**). The changes in spleen pathology of the three groups are shown in **Figure 5**, and the changes in other tissue weight over body weight (liver, spleen, right and left kidneys, and lung) are shown in **Supplementary Figure 3**. The spleen-to-body weight ratio showed significantly higher percentages in the vaccinated groups (vaccine A; *p = 0.025 and vaccine-B; *p = 0.042) compared with the SARS-CoV-2-infected group (**Figure 5A**). The spleen showed to be enlarged under infectious conditions due to the increased immune cell proliferation and differentiation against infected pathogens such as SARS-CoV-2. Thus far, the severity of the damage after infection or injury is mainly measured by the white pulp atrophy rather than spleen enlargement, whereas the atrophy lesion in the splenic white pulp was shown to be increased in the vaccine A-administered group with no significant difference in the vaccine B-and SARS-CoV-2-infected groups (**Figures 5B, C**). Furthermore, small intestine goblet cells are known for their participation in the immune response; however, increasing the number of these cells indicated worsening of the case and mucus hypersecretion will result in goblet cell hyperplasia (37). In **Figure 6**, the histopathological changes in terms of goblet cell number of the three mice were evaluated. As the results showed, the vaccine B-administered group represents a statistically significant low number of goblet cell in villi (*p = 0.027), representing a healthier intestine among the three groups.

## 4 Discussion

Effective vaccine development has been a critical concern throughout the last pandemic caused by SARS-CoV-2. The need emerged from the start of this pandemic in late 2019, which encouraged many companies to work on a solution in a highly accelerated time frame (32, 38–42). Despite the several vaccines produced, there is still a need for more effective and safe vaccines against COVID-19 as the causing virus, SARS-CoV-2, continues to spread worldwide (33). It is true for a number of vaccines that it was successful in developing a vaccine in record time, yet many challenges remain to be solved to overcome the COVID-19 pandemic emergency (11, 34).

Here, in this study, we were able to select potential spike antigens after evaluation of a list of antigens using infected and vaccinated human sera. In addition, we compared a single antigen versus a mixture of antigens to assess, which could be the best vaccination strategy. Moreover, our results demonstrated the mixture of antigens; vaccine B has promising properties for the development of a vaccine against SARS-CoV-2. In developing peptide-based vaccines, choosing the appropriate epitopes/antigens is the most critical step. The spike (*S*) protein is a crucial protein on the SARS-CoV-2 viral surface as it binds to the host cell surface and mediates the invasion (43, 44). Thus, it is the foremost target of the majority of the anti-COVID-19 vaccines currently offered. Therefore, by narrowing a pool of spike antigens, we aimed to maximize the effectiveness of vaccine candidates. We started with various antigens/epitopes reaching four potential antigens by evaluating the binding of antigens with neutralizing antibodies in the sera of vaccinated and infected patients; these four selected antigens were subjected to further *in vivo* analysis.

Next, we want to evaluate the impact of the number of antigens per vaccine, so we designed two vaccines, which are vaccine A, which is composed of one antigen (Ag1), and vaccine B, which is composed of an equal mixture of the four selected antigens (Ag1–Ag4). Our results demonstrated that vaccine B with a mixture of antigens provides better outcomes in terms of viral load and histopathological changes than that of a single antigen, vaccine A. Moreover, when we validated the formation of antibodies by titration assay for both vaccinated groups (**Figure 2B**), as expected, the vaccine-B group showed binding with all four antigens, especially with Ag4. The vaccine-A group revealed binding with Ag1 as expected but also showed a cross-reactivity with Ag3. Interestingly, the vaccine-A group showed high affinity binding to Ag3, although vaccine A does not contain Ag3. Moreover, the OD_450_ of three mice (A3–A5) for Ag3 was slightly higher than Ag1 in this group, indicating a cross-reactivity occurrence that might be developed by a possible shared epitope structure resulting from a short stretch of amino acids. Furthermore, it is interesting that vaccine-B group mice did not develop an antibody against Ag3, although vaccine B contains Ag3. These data indicate that within a mixture of antigens, some antigens tend to be more visible to the immune system than others.

On the other hand, vaccine B owns four polypeptides of spike protein that interestingly have some amino acid stretches mentioned for their immunogenicity in previous studies (underlined in **Table 1**). The epitope/motif QCVNLTTRT in Ag1 was predicted for its antigenicity that was examined for the mutational events and thus, being a highly potential vaccine candidate, was already validated by our assessment (45). In addition, an epitope KPFERDISTEIYQAG STPCNGVEGFNCYFPLQS, found within Ag1 and Ag2, was recognized earlier among other epitopes that induce long-term immunity (46). However, Ag2 and Ag4 present higher titration results indicating higher binding with the formed antibodies (**Figure 2B**). Yet, all four antigens as one vaccine offer the overall outcome. More detailed investigations are needed to evaluate which antigens produce higher immunity.

In conclusion, it has been well established that vaccination is the most effective strategy for controlling and eradicating infectious diseases. In this study, we sought to evaluate the efficacy of two proposed SARS-CoV-2 vaccines, vaccine A and vaccine B, in providing protection against infection with the virus. Vaccine A was composed of a single SARS-CoV-2 polypeptide derived from the viral spike protein, whereas vaccine B consisted of multiple polypeptides also derived from the spike protein. Upon administering the vaccines to a group of study subjects, we observed that vaccine B was able to attenuate histopathological changes in organs and provided superior protection against SARS-CoV-2 infection compared with vaccine A, as shown in **Figures 4****–** **6**. These findings suggest that multivalent spike protein-based vaccines may be more effective at inducing immunity against SARS-CoV-2. To the best of our knowledge, this is the first study to compare the efficacy of mono- and multi-peptide vaccine formulations against SARS-CoV-2. Based on the promising results of this study, further investigation into the use of multi-peptide-based vaccines such as vaccine B may hold promise as a potential candidate for the development of an effective COVID-19 vaccine.

## Data availability statement

The original contributions presented in the study are included in the article/**Supplementary Material**. Further inquiries can be directed to the corresponding authors.

## Ethics statement

All the animal experiments were approved by the Institutional Animal Care and Use Committee (IACUC) at Konkuk University. Written informed consent was obtained from the owners for the participation of their animals in this study.

## Author contributions

YH, S-MS, and SK designed the study, analyzed the data, and performed the experiments. SS, JH, E-SY, and N-WK, performed the experiments. YH, C-SS, HJ, H-YP, YL, K-CS, and S-YH analyzed the data. Funding acquisition: H-YP, JS, Y-KC, and SHK. YH and S-MS examined the data. YH edited the manuscript. Y-KC and SHK designed the study, supervised the project, and wrote the manuscript.
