# Supplementary material for: COVID-19 spike polypeptide vaccine reduces the pathogenesis and viral infection in a mouse model of SARS-CoV-2
Source: Front Immunol. 2023 Mar 3;14:1098461. doi: 10.3389/fimmu.2023.1098461 (PMC10020603; doi:10.3389/fimmu.2023.1098461)
Supplement: Supplementary file 1 [file DataSheet_1.docx]

**COVID-19 spike polypeptide vaccine reduces pathogenesis and viral infection in mouse model of SARS-CoV-2**

Running title: COVID-19 spike polypeptide vaccine


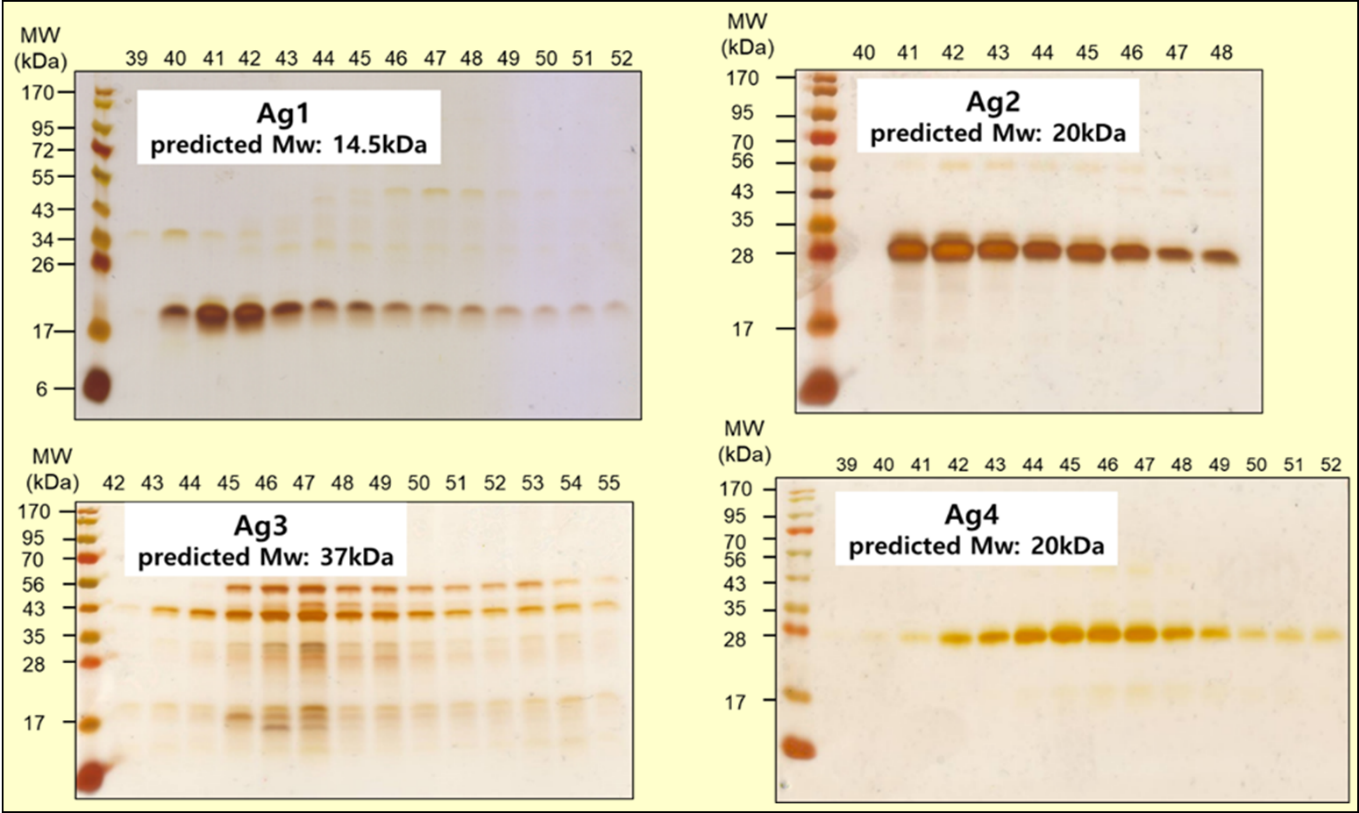


**Supplementary Figure 1.** 10% SDS-PAGE silver stained of the recombinant antigens were purified by two-steps, a mini-Talon and HPLC. HPLC fraction number was indicated at the top of each lane. An expected molecular weight of antigen was written in SDS-PAGE silver staining. The amino acid sequence of four antigens (Ag1 – Ag4) was shown in **Table 1**.


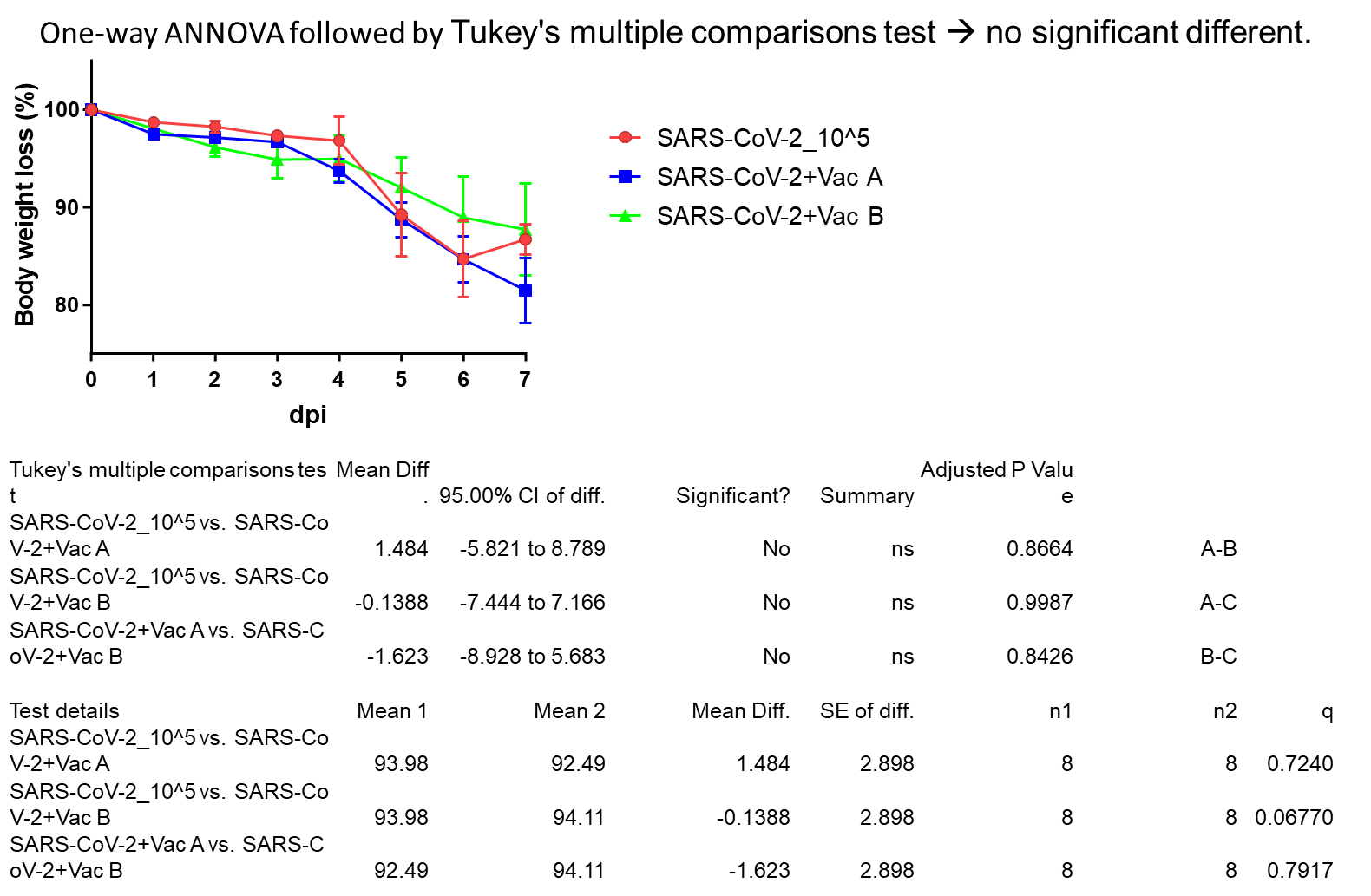


**Supplementary Figure 2.** Body weight loss percentage from day 0 up to day 7 post infection for the three groups of mice; vaccine-A, vaccine-B with SARS-CoV-2 infected group, and only SARS-CoV-2 infected group (each n=5), means with SD are presented and there was not significant among groups.


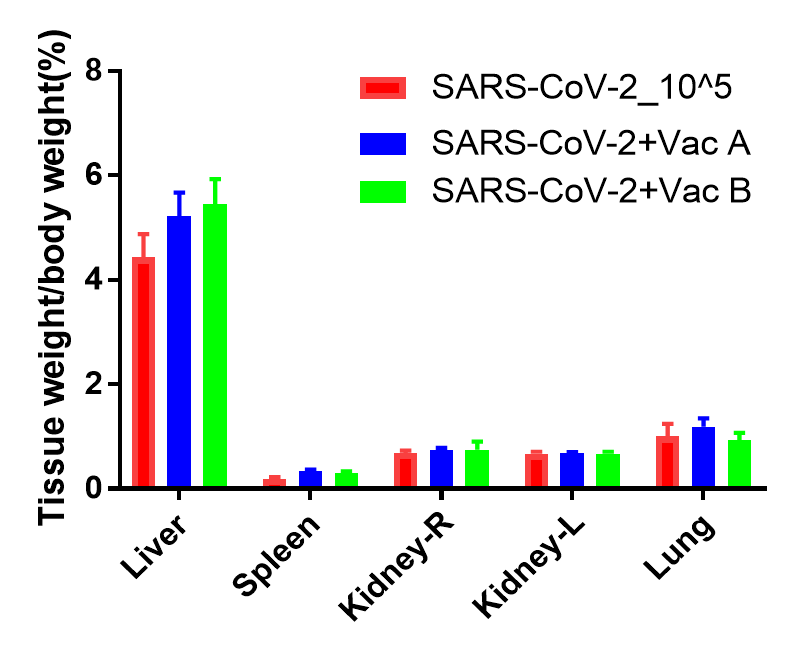


**Supplementary Figure 3.** Ratio of organ wight over body weight represent as percentage in liver, spleen, right and left kidneys, and lung of the three groups of mice; vaccine-A, vaccine-B with SARS-CoV-2 infected group, and only SARS-CoV-2 infected group (each n=5), means with SD are presented and there was not significant among groups.
